# Multiple Effects of SGLT2 Inhibitors

**DOI:** 10.31662/jmaj.2024-0229

**Published:** 2024-09-20

**Authors:** Hajime Nagasu

**Affiliations:** 1 Department of Nephrology and Hypertension, Kawasaki Medical School, Okayama, Japan

**Keywords:** SGLT2 inhibitor, hyperkalemia, acute kidney injury, anemia, chronic kidney disease

In Japan, SGLT2 inhibitors will be covered by insurance for chronic kidney disease in 2019 and are now the mainstay of chronic kidney disease treatment. Under normal conditions, the glucose glomerular filtration rate is approximately 180 g per day. Most of this is reabsorbed in the proximal tubules and is not excreted in urine. Reabsorption capacity is possible up to about 375 mg/min in healthy individuals. By contrast, at high blood glucose levels above this level, urinary glucose appears. SGLT plays a major role in glucose reabsorption in the proximal tubules. In particular, SGLT2 is predominantly expressed in the proximal tubules, and its expression is further increased in patients with type 2 diabetes; even when SGLT2 is completely inhibited, glucose can be reabsorbed by SGLT1 at a rate of up to 120 g/day. Therefore, the risk of hypoglycemia with SGLT2 inhibitors is low.

Several clinical studies have demonstrated the renoprotective effects of SGLT2 inhibitors. In diabetic kidney disease, the Canagliflozin and Renal Outcomes in Type 2 Diabetes and Nephropathy (CREDENCE) ^(1)^ study demonstrated its efficacy. Subsequently, the Dapagliflozin and Prevention of Adverse Outcomes in Chronic Kidney Disease (DAPA-CKD) trial ^(2)^ included chronic kidney disease with and without type 2 diabetes. The DAPA-CKD trial demonstrated the efficacy of SGLT2 inhibitors in patients with and without type 2 diabetes. This study is substantial because it is the first study of an SGLT2 inhibitor in chronic kidney disease. The enrolment criteria were eGFR (25-75 mL/min/1.73 m^2^) and ACR (2,00-5,000 mg/g), a slightly lower renal function group than in CREDENCE. The primary composite outcome was a sustained reduction in eGFR ≥50%, end-stage renal failure (defined as initiation of renal replacement therapy or eGFR <15 mL/min/1.73 m^2^ at assessment after 28 days), renal disease-related death, and cardiovascular death. The results showed a significant reduction in the occurrence of primary composite outcomes in the Dapagliflozin group compared with the placebo group (hazard ratio [HR], 0.61; 95% CI, 0.51-0.72; P < 0.001). Furthermore, the HR for the Renal Specific Composite Outcome in the Dapagliflozin-treated group (vs. placebo group) was 0.56 (95% CI 0.45-0.68; P < 0.001), and the HR for the composite endpoint of cardiovascular death and heart failure hospitalization was 0.71 (95% CI, 0.55-0.92; P = 0.009). In the stratified analysis of this study, the primary composite outcome was also reduced in the Dapagliflozin-treated group in patients with non-Type 2 diabetes (HR, 0.63; 95% CI, 0.52-0.79) in patients with Type 2 diabetes compared with an HR of 0.63 (95% CI, 0.52-0.79) in non-Type 2 diabetics had a lower HR of 0.50 (95% CI, 0.35-0.72).

By contrast, SGLT2 inhibitors have been shown to have various secondary effects. They have been shown to increase hematocrit ^(3)^, lower uric acid and prevent gout attacks ^(4)^, and prevent the onset of hyperpotaschaemia ^(5)^ and inhibition of the development of acute kidney injury ^(6)^.

SGLT2 inhibitor treatment initially causes an initial dip. Therefore, the risk of acute kidney injury is a concern with SGLT2 inhibitor administration, as with renin angiotensin system inhibitors. Analyses using US Medicare data have reported comparisons between SGLT2 and DPP4 inhibitors or SGLT2 inhibitors and GLP1analog. This study was a huge cohort, with 68,130 patients in the SGLT2 inhibitor-DPP4 inhibitor comparison and 71,477 patients in the SGLT2 inhibitor-GLP1analog comparison after propensity score matching. The results showed a considerable reduction in the incidence of AKI in the SGLT2 inhibitor group in both comparisons.

Although SGLT2 inhibitors have a wide variety of effects, the factors are involved in their respective effects remain unclear. This study focuses on this point ^(7)^ and evaluated the impact of SGLT2 inhibitor treatment when stratified by eGFR60. In patients with normal eGFR, no correlation is observed between the previous value of hematocrit and the effect on anemia. By contrast, in the group of patients with reduced eGFR, the effect of the increase in anemia was higher than in the group of patients with low hematocrit. This indicates that the effect of SGLT2 inhibitors for anemia is not owing to simple fluid changes. Conversely, GFR levels are unaffected with regard to uric acid changes.

Further investigation of various factors is believed to lead to a better understanding of the mechanisms of the condition and the efficacy of the drugs.

## Article Information

### Conflicts of Interest

None
